# Broadband beamforming compensation algorithm in CI front-end acquisition

**DOI:** 10.1186/1475-925X-12-18

**Published:** 2013-02-27

**Authors:** Yousheng Chen, Qin Gong

**Affiliations:** 1 Department of Biomedical Engineering, Tsinghua University, Beijing 100084, P.R. China

## Abstract

**Background:**

To increase the signal to noise ratio (SNR) and to suppress directional noise in front-end signal acquisition, microphone array technologies are being applied in the cochlear implant (CI). Due to size constraints, the dual microphone-based system is most suitable for actual application. However, direct application of the array technology will result in the low frequency roll-off problem, which can noticeably distort the desired signal.

**Methods:**

In this paper, we theoretically analyze the roll-off characteristic on the basis of CI parameters and present a new low-complexity compensation algorithm. We obtain the linearized frequency response of the two-microphone array from modeling and analysis for further algorithm realization.

**Realization and results:**

Linear method was used to approximate the theoretical response with adjustable delay and weight parameters. A CI dual-channel hardware platform is constructed for experimental research. Experimental results show that our algorithm performs well in compensation and realization.

**Discussions:**

We discuss the effect from environment noise. Actual daily noise with more low-frequency energy will weaken the algorithm performance. A balance between low-frequency distortion and corresponding low-frequency noise need to be considered.

**Conclusions:**

Our novel compensation algorithm uses linear function to obtain the desired system response, which is a low computational-complexity method for CI real-time processing. Algorithm performance is tested in CI CIS modulation and the influence of experimental distance and environmental noise were further analyzed to evaluate algorithm constraint.

## Background

Over 120,000 recipients with severe hearing loss currently use a cochlear implant (CI) to improve their sound sensation [1]. Cochlear implant recipients generally require higher signal to noise ratios (SNRs) than normal listeners to obtain similar speech recognition [2]. In noisy environments, such as the cocktail party situation, the practical performance of the CI device is dramatically weakened [3,4]. Microphone array technologies, which use multiple sensors to obtain additional spatial information for noise suppression, have been applied in hearing aids (HAs) [5-8]. Given the size limits in CI products, the dual-channel array is most suitable for front-end signal acquisition. Cochlear implants are similar to HAs in their front-end hardware and speech-enhancement requirements. Moreover, CIs borrow various noise-suppression methods from HA applications. Many theoretical and experimental studies of array speech enhancement have been performed to improve CI speech recognition [9-11]. A dual-microphone CI was recently designed by Cochlear Ltd. to improve the front-end SNR. However, the clinical CI device is still equipped with a single omnidirectional microphone, and no microphone array-based method is applied in daily usage for the CI device.

Microphone array methods [12,13] add spatial information to the algorithms and are especially effective in suppressing directional noise. These array-beamforming methods include fixed [14,15] and adaptive beamformers [16-20]. Present complicated algorithms combine beamforming technology with single-channel signal-filtering and noise-suppression methods [21-23]. The delay and sum beamforming method, especially with two microphones, is more suitable for application in the CI device, given its appropriate size constraints and low complexity in real-time processing [24].

Microphone array technologies are very effective to narrowband signals, whereas speech is a broadband signal. The direct application of narrowband technologies to acoustic and phonic fields may result in low frequency roll-off. Studies have reported the practical implications of low frequency roll-off for HA devices [25], comparing sensatory performance results between situations with and without compensation for low frequency roll-off. Theoretically, slopes for the first- and second-order low frequency roll-off are 6 and 12 dB/octave, respectively [1].

A solution for the low frequency roll-off is gain adjustment in each sub-band. Corresponding gains need to compensate for the energy loss, and the sub-band signal is readjusted to be the same with the omnidirectional microphone [26]. A first-order differential low-pass filter is conventionally applied [17,19] for compensation of the low frequency roll-off. However, these methods cannot accurately compensate the desired gains for the low frequency roll-off. Increased focus has been given to broadband beamforming [27,28]. Post-filter technology uses maximum likehood filter for spectral shaping and noise suppression [29]. However, these methods are very complicated, require large computational complexity, and exceed the capabilities of current CI speech processors for portable application.

We use the array method for CI speech enhancement and further need to compensate for the signal distortion introduced from the low frequency roll-off. For a simple dual-microphone device with a delay parameter, we previously proposed the normalized beamforming algorithm [30] based on the Taylor approximation. However, this method is not applicable for a general array with delay and weight parameters.

In this paper, we theoretically analyze the low frequency roll-off feature under different parameters. A dual-microphone hardware platform, based on actual CI parameters, was constructed to obtain practical experimental results. From theoretical analysis and experimental observation of the roll-off, we propose a novel compensation algorithm for the low frequency roll-off with adjustable delay and weight parameters. The proposed algorithm can accurately compensate the signal distortion and requires very few calculations. Further testing of this adjustment method in the CI speech strategy revealed the efficiency and applicability of the proposed compensation algorithm.

## Methods

### Dual-microphone system in CI front-end

**Figure 1:**
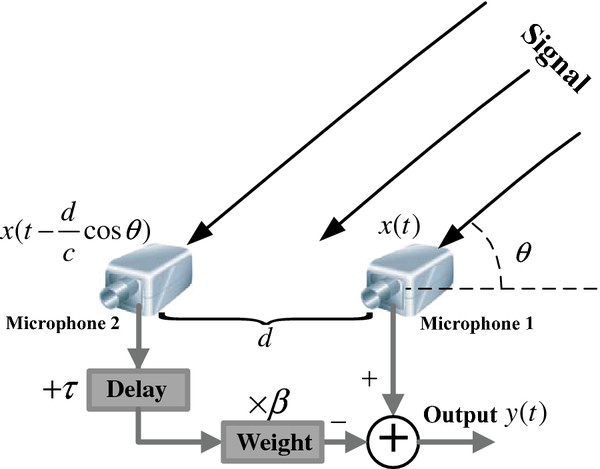
Sketch of delay-and-subtract method in the dual-microphone system.

Given the size constraint of CI devices and the limited calculation capability in the speech processor, the dual-microphone array is most suitable for use in front-end signal acquisition. Figure 1 presents the system sketch for a dual-microphone array with delay and weight parameters based on the “delay-and-subtract” method. According to practical requirements, the inter-microphone distance is about 1 cm. Dual-channel signals recorded by these 2 microphones are delayed and subtracted to yield the desired directional output signal.

The delay-and-subtract method is one of the most effective technologies in size-constraint device, hearing aid and cochlear implant etc. In Figure 1, the two microphones each record the signal, d is the inter-microphone and c is the speed of sound in air. If we assume that the recorded signal in Microphone 1 is x(t), then the recorded signal in Microphone 2 is asynchronous due to the spatial difference, with a additional time delay $\frac{d}{c}\text{cos}\theta$. This signal is delayed and weighted with the corresponding parameters τ and β to yield a new signal, which is combinated with the signal in Microphone 1 to obtain the directional output y(t). The system magnitude response is given by Eq. (1).

(1) $$\left|H\left(e^{j\omega }\right)\right|=\left|1-\beta e^{-2\mathrm{j\pi}f\left(\frac{d}{c}\text{cos}\theta +\tau \right)}\right|$$

where the parameters f corresponds to the narrowband frequency. For a different orientation θ, the system response is also different. The algorithm delay τ corresponds to the beam pattern (dipolar pattern: τ = 0; supercardiod pattern: τ = 0.342d/c; cardioids pattern: τ = d/c) [31].

A fixed value of the delay τ can yield a set of similar beam patterns. The magnitude response in Eq. (1) is based on a specified narrowband frequency to obtain the directional beams. Therefore, the system response is a function of the signal frequency f, and the use of different frequencies results in different magnitude responses.

### Low frequency roll-off characteristic

**Figure 2:**
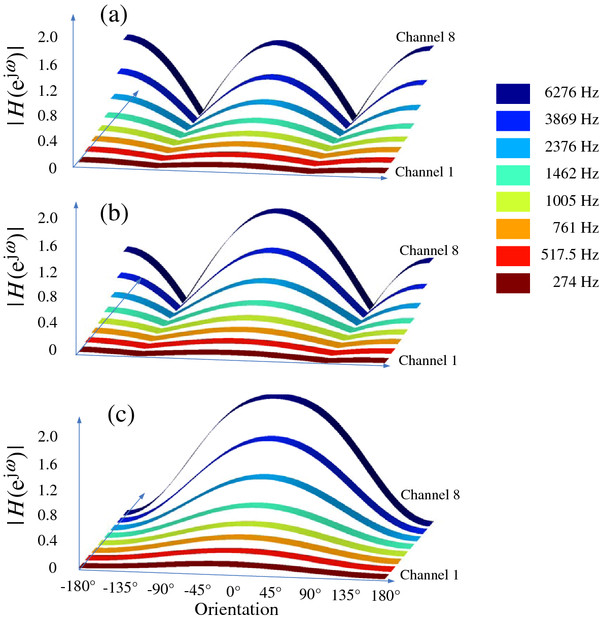
System responses based on different frequencies for (a) dipolar, (b) supercardioid, and (c) cardioid beam patterns.

We use the actual parameters of a CI device, d = 0.01 m and c = 340 m/s, in the theoretical analysis. We initially analyze a simplified situation, in which the weight parameter β is chosen to be 1, and the 2 microphones are equally weighted. Figure 2 shows the dipolar, supercardiod, and cardioid beam patterns for different frequencies in this situation.

Figure 2 describes changes in the dual-microphone beams for different frequencies (correspond the center frequencies of 8-channel CI filter bank, shown in Table 1).

**Table 1:** Frequency parameters in 8-channel CI filter bank

	**Band edge (Hz)**	**Center frequency (Hz)**
Channel 1	[156, 396]	274
Channel 2	[396, 639]	517.5
Channel 3	[639, 883]	761
Channel 4	[883, 1127]	1005
Channel 5	[1127, 1797]	1462
Channel 6	[1797, 2955]	2376
Channel 7	[2955, 4783]	3869
Channel 8	[4783, 7769]	6276

The system magnitude response in Eq. (1) is a function of signal frequency. In each beam pattern (dipolar, supercardiod, and cardioid), the system response increases as the corresponding frequency increases. Because speech is a broadband signal including various frequencies, the corresponding beams are not consistent with each other. In addition, the system response for low-frequency beams is smaller than that for high-frequency ones. Observed from these panels, the main lobe and side lobe amplitude both increase at higher frequency. Therefore, we observe low frequency roll-off in the broadband application, which will introduce distortion to the desired speech.

We can theoretically analyze the reason of low frequency roll-off. To simply the analysis, let β = 1, and the correspond response simplified in Eq. (2).

(2) $$\begin{array}{cc} \left|H\left(e^{j\omega }\right)\right|=2\left|\text{sin}\left(\mathrm{\pi f}\left(\frac{d}{c}\text{cos}\theta +\tau \right)\right)\right|\approx 2\mathrm{\pi f}\left(\frac{d}{c}\text{cos}\theta +\tau \right) & \left(\mathrm{if}\;f\to 0\right) \end{array}$$

When signal frequency is low, the system response is approximately proportional to f. Therefore, low frequency corresponds to small response.

**Figure 3:**
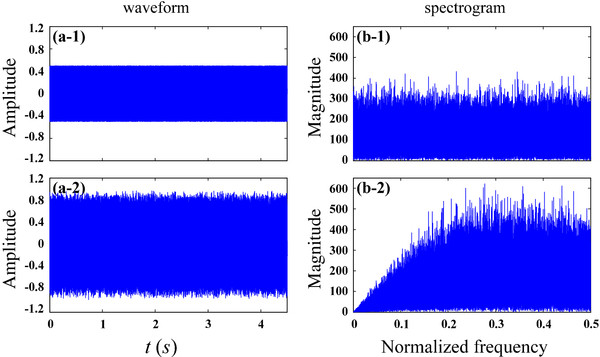
Waveform of the original white noise signal (panel a-1) and the first-order differential output signal (panel a-2); and the corresponding spectrum are presented in panels of (b-1) and (b-2) respectively.

As an example, this roll-off feature can easily be observed when we use white noise as testing signal, shown in Figure 3.

The spectrogram of white noise contains with information from all frequencies. Correspondingly, the first-order output will noticeably weaken the low-frequency signal in (b-2).

Based on Eq. (1), we can deduce the following inequality.

(3) $$\begin{array}{c} \left|1-\beta e^{-2\mathrm{j\pi}f\left(\frac{d}{c}\text{cos}\theta +\tau \right)}\right|=\left|1-\beta \text{cos}\left(-2\pi f\left(\frac{d}{c}\text{cos}\theta +\tau \right)\right)\right)\\ \;-\left(j\beta \text{sin}\left(-2\pi f\left(\frac{d}{c}\text{cos}\theta +\tau \right)\right)\right|\\ \;=\sqrt{1+\beta^{2}-2\beta \text{cos}\left(-2\pi f\left(\frac{d}{c}\text{cos}\theta +\tau \right)\right)}\geq \sqrt{1+\beta^{2}-2\beta }\\ \;=\left|1-\beta \right| \end{array}$$

It indicates that, for the situation of β = 1, the system response will approach zero, which corresponds to frequency f = 0. Therefore, the amplitude response of the low-frequency signal is smaller and will approximate to zero.

### Parameter analysis

**Figure 4:**
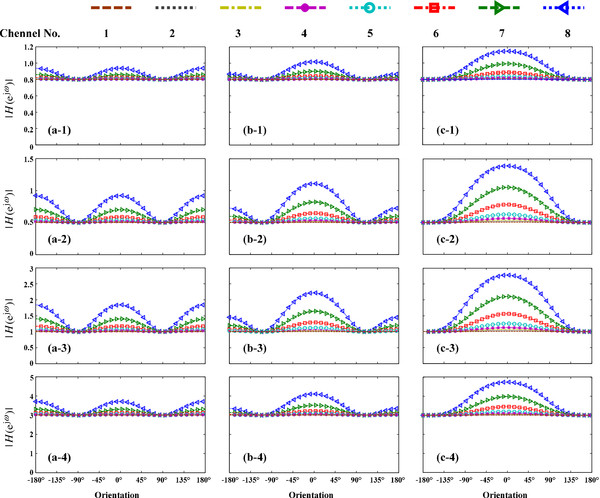
**Effect of the weight parameters on the system response for (a) dipolar, (b) supercardioid, and (c) cardioid beam patterns, with corresponding weight parameters**. Panels **a-1**, **b-1**, **c-1**: *β* = 0.2; **a-2**, **b-2**, **c-2**: *β* = 0.5; **a-3**, **b-3**, **c-3**:* β* = 2; **a-4**, **b-4**, **c-4**: *β* = 4.

The most common situation uses a weight parameter of β = 1. Use of different weights in the algorithm will change the corresponding beam patterns. We analyze beam features with delay times of τ = 0, 0.342d/c, and d/c (Figure 4).

Figure 4 describes the influence of different weights on the system response (Channel No. 1, 2, 3, 4, 5, 6, 7 and 8 correspond to 274, 517.5, 761, 1005, 1462, 2376, 3869 and 6276 Hz respectively). For β = 1, the aforementioned analysis indicates that the low-frequency response will approach zero. For β ≠ 1, the system response in the low-frequency band does not approach zero, but approximates to a specific amplitude. For β = 0.2, the low-frequency band approaches approximately 0.8. For β = 0.5, 2, and 4, the corresponding low-frequency responses are approximately 0.5, 1, and 3, respectively, consistent with the theoretical analysis of |*H*(***e***^***j****ω*^)|_min_ = |1 - *β*| in Eq. (3).

In the previous analysis, the low-frequency response does not approach zero, but low frequency roll-off and response distortion are observed. In each panel, the system response increases with increasing signal frequency. In Figure 4, a-1, a-2, a-3, and a-4 refer to derivative patterns based on the standard dipolar beam (τ = 0). In the range of ± 180°, we observe the same amplitude-changing feature, such that the high-frequency band increases. The dipolar pattern in Figure 2(a) shows zero-response values (Nulls) at the ±90° orientations. In contrast, the derived dipolar beam (β ≠ 1) in the 4 panels of Figure 4(a) shows Minimal values (Mins) rather than Nulls at ±90°. For τ = 0.342d/c and d/c, the corresponding Mins are obtained at ±110° and ±180°, respectively.

**Figure 5:**
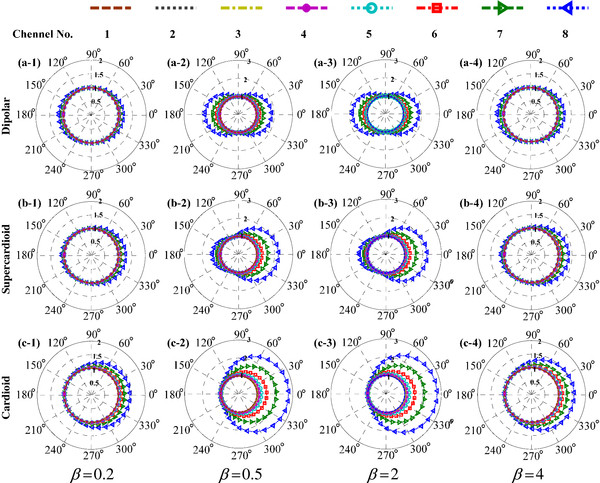
**Normalized beam patterns for (a) dipolar, (b) supercardioid, and (c) cardioids, with corresponding weight parameters. **Panels **a-1**, **b-1**, **c-1**: *β* = 0.2; **a-2**, **b-2**, **c-2**: *β* = 0.5; **a-3**, **b-3**, **c-3**: *β* = 2; **a-4**, **b-4**, **c-4**: *β* = 4.

In Figure 4, the system response is located at different amplitude ranges for different weights. Because the overall amplitude levels are different, low frequency roll-off cannot be quantitatively observed directly from the figure. To compare each roll-off characteristic on a unified standard, we normalize system responses at 0 Hz to be the same. For a fixed weight, the amplitude at each orientation differs from the others. Therefore, we need to normalize the average amplitude from -180° to 180° to be 1. The 0 Hz frequency is an infinitesimal case of low frequency, as the corresponding frequency response approximates to |1 - *β*| (shown in Eq. 3). Figure 5 shows the normalized system responses.

Figure 5 describes the beam patterns on the basis of the response normalization at 0 Hz. In each panel, the system response increases with increasing frequency, but the magnitude of the increase differs for different parameters. After normalization, for β = 0.5 (a-2, b-2, and c-2) and β = 2 (a-3, b-3, and c-3), the system responses can be more easily distinguished and the high-frequency response increases more. In contrast, for β = 0.2 (a-1, b-1, and c-1) and β = 4 (a-4, b-4, and c-4), the system response changes more smoothly. The situation with β > 1 corresponds to a larger gain added to Microphone 2, and β < 1 corresponds to a larger gain added to Microphone 1. Similar to the previous comparison, when the weight approaches 1, the high-frequency beams grow more rapidly, which results in more low frequency roll-off. When the weight parameter is far from 1 (≪ 1 or ≫ 1), the roll-off and corresponding distortion are less than the previous ones.

The system response for β < 1 can be given with the following equation transform.

(4) $$\begin{array}{c} \left|1-\beta_{1}e^{-2\mathrm{j\pi}f\left(\frac{d}{c}\text{cos}\theta +\tau \right)}\right|=\left|1-\beta_{1}e^{-2\mathrm{j\pi}f\left(\frac{d}{c}\text{cos}\theta +\tau \right)}\right|\cdot \left|e^{2\mathrm{j\pi}f\left(\frac{d}{c}\text{cos}\theta +\tau \right)}\right|\\ \;=\beta_{1}\left|1-\frac{1}{\beta_{1}}e^{2\mathrm{j\pi}f\left(\frac{d}{c}\text{cos}\theta +\tau \right)}\right|=\beta_{1}\left|1-\frac{1}{\beta_{1}}e^{-2\mathrm{j\pi}f\left(\frac{d}{c}\text{cos}\theta +\tau \right)}\right| \end{array}$$

The equivalent equation in Eq. (4) indicates that the system response for β_1_ < 1 corresponds to the case of $\beta_{2}=\frac{1}{\beta_{1}.}>1$, with only an additional gain paramete, β_1_. Therefore, the normalized system response presents identical roll-off features for both situations. Equation (4) indicate that the weight value has reciprocal and symmetrical features; thus, we only need to consider the case of β < 1.

## Realization and results

### Low frequency roll-off in dual-channel CI platform

**Figure 6:**
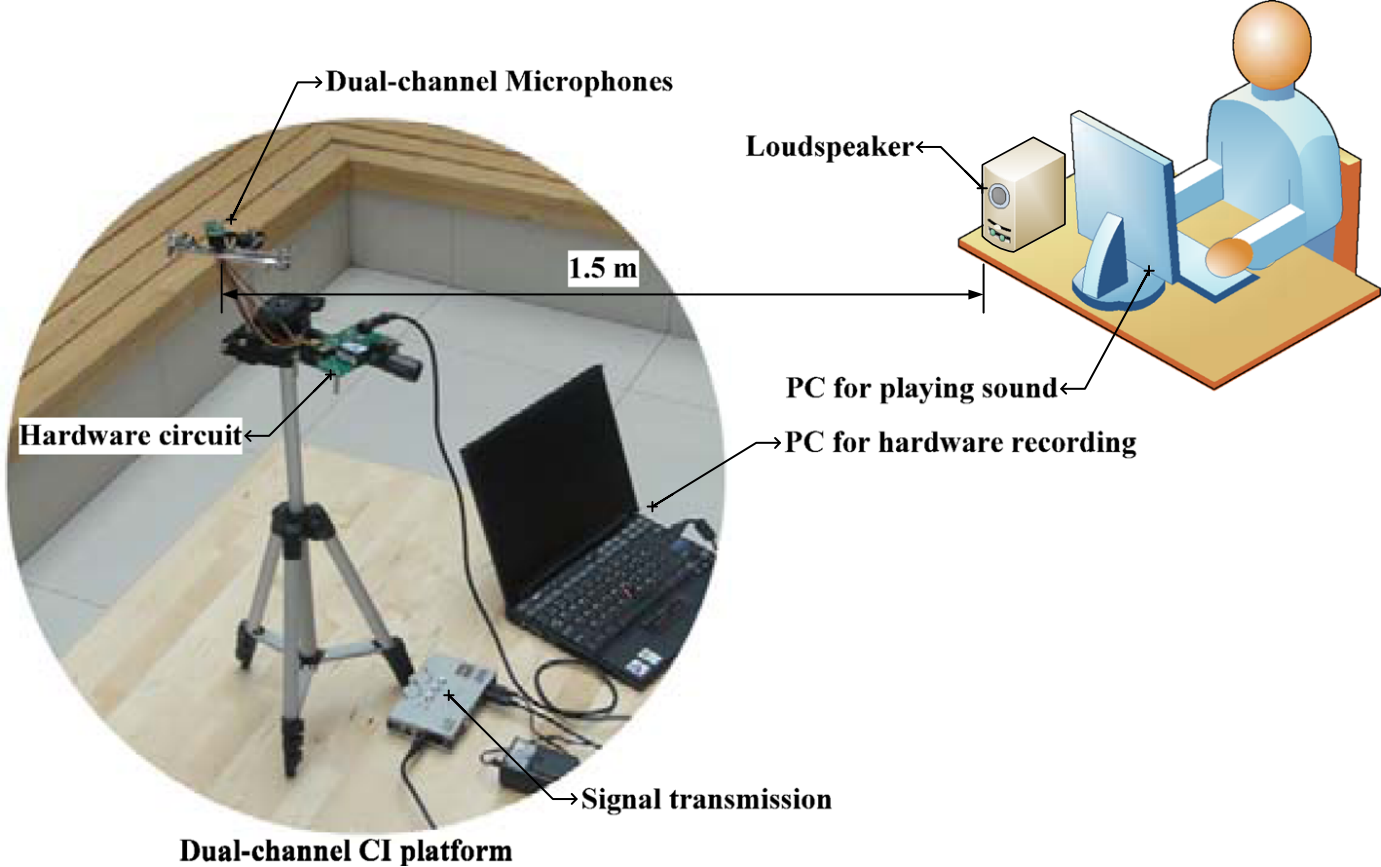
Dual-channel CI front-end platform.

In this paper, we develop a dual-channel hardware platform, based on actual CI size constraints and design requirements, for signal acquisition and algorithm analysis. The hardware system includes microphone modules, a signal acquisition circuit, signal transmitting device, computer, and accessory devices (holder frame, etc.), as shown in Figure 6.

The inter-microphone distance is adjusted to be 1 cm, and the loudspeaker is 1.5 m apart from the hardware. The experiment is conducted in an ordinary room (10 m × 8 m × 3.5 m) with a reverberation time of about 400 ms.

**Figure 7:**
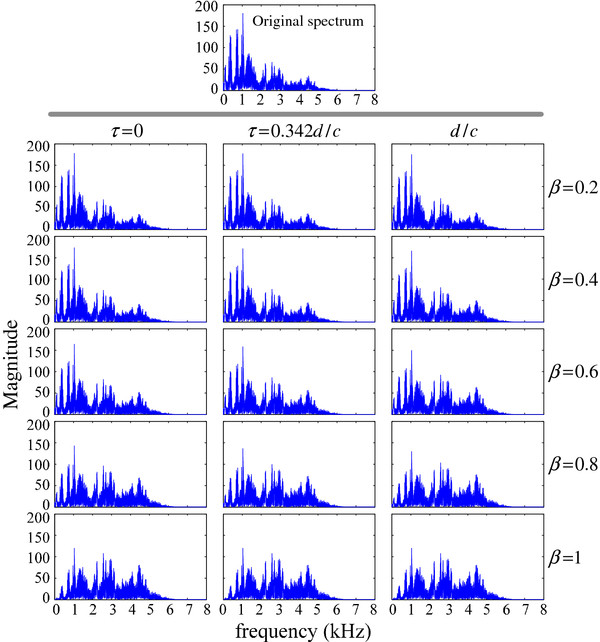
Signal spectrums for different delay times and weights.

As the experiment begins, the loudspeaker plays a voice recording of a speaker reading a paragraph of English material (Native American English). Two microphones record this signal from the loudspeaker, and the collected signal is amplified and filtered by the hardware circuit. The USB sound card transfers the analog signal to digital signal, which is transmitted to the computer. The data are stored on a PC hard disk for further analysis. To simplify the analysis of low frequency roll-off with a unified standard, an additional gain is added to the output signal to maintain a consistent input-output energy. On the basis of the previous theoretical analysis, the weight only needs to be ≤1. We choose the weight parameter to be 0.2, 0.4, 0.6, 0.8, or 1 and the delay parameter τ to be 0, 0.342d/c, or d/c, to analysis the practical spectrums of the recorded signal by the hardware (Figure 7).

Figure 7 shows signal spectra for different delay times and weights. To simplify the analysis, the corresponding spectrum at each panel is normalized to maintain the same energy with the original signal. The spectrum of the original signal is shown for a detailed comparison of the low frequency roll-off. For a fixed delay time, as the weight increases, the high-frequency response strengthens and the low-frequency response weakens. For a weight parameter of 1, the low frequency roll-off is most noticeable. For this speech signal, after energy normalization, signals in the band between 0 and 1800 Hz are relatively weak, whereas the corresponding signals are relatively strong in the high-frequency band (>1800 Hz). The delay parameter also influences the spectrum distribution, although this effect is not as obvious as the influence of the weight parameter.

### Compensation algorithm

**Figure 8:**
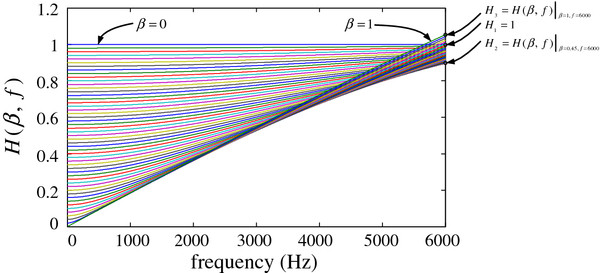
System-response function for different weights and frequencies.

We next attempt to compensate for the signal distortion based on the low frequency roll-off feature. For different weight and frequency parameters, the system response is a function of β and f, *H* (***e***^***j****ω*^) = *H* (*β*,  *f*). Using a weight range of 0 ≤ β ≤ 1 (similar to the previous section), the system responses for a signal between 0 and 6000 Hz are shown in Figure 8 (dipolar-based parameter of τ = 0).

Figure 8 presents the system responses for different weights and frequencies, in which the weight is increasing at an interval of 0.02. The response curves are equally spaced, with good linearity (especially in the high-frequency band). We use (ƒ, H (*β*, ƒ)) to describe points in the 2-dimensional plane for the f-H function. For points on the left vertical axis (corresponding to f = 0), the system responses are equally space distributed. For a fixed weight β, the response function *H* (*β*,  *f*)|_*f* = 0_ = 1 - *β*, such that the coordinate of the left endpoint in the f-H function is (0, 1–β). For f = 6000 Hz, the right endpoints in the response curves are also equally spaced, but with a turning point of H_2_ corresponding to β = 0.45.

From Figure 8, we calculate three response values: *H*_3_ = *H* (*β*,  *f*)|_*β* = 1,*f* = 6000_ = 1, *H*_2_ = *H* (*β*,  *f*)|_*β* = 0.45,*f* = 6000_, and *H*_3_ = *H* (*β*,  *f*)|_*β* = 1,*f* = 6000_. For the case of 0 ≤ β. ≤ 0.45, the system response at f = 6000 Hz decreases in an equally spaced manner from H_1_ to H_2_. For the case of 0.45 < β. ≤ 1, the system response increases in an equally spaced manner from H_2_ to H_3_. Consequently, we obtain the following system response at the frequency f = 6000 Hz.

(5) $$H\left(\beta ,\;f\right)|{}_{f=6000}=\{\begin{array}{cc} 1-\frac{\beta }{0.45}\left(1-H_{2}\right),\; & 0\leq \beta \leq 0.45\\ H_{2}+\frac{\beta -0.45}{0.55}\left(H_{3}-H_{2}\right), & 0.45<\beta \leq 1 \end{array}$$

The previous analysis indicates the approximate linearity of the system response. To reduce the computational complexity in CI devices, we linearize the f-H function. We use the curve endpoints (0, β) and (6000, *H* (*β*,  *f*)|_*f* = 6000_) to yield the approximate system response H_eva_ (β, ƒ), as shown in Eq. (6).

(6) $$H_{\text{eva}}\left(\beta ,\;f\right)=\{\;\begin{array}{c} \;1-\beta +\frac{\left(1-\frac{\beta }{0.45}\left(1-H_{2}\right)\right)-\left(1-\beta \right)}{6000}f,\;0\leq \beta \leq 0.45\\ 1-\beta +\frac{H_{2}+\frac{\beta -0.45}{0.55}\left(H_{3}-H_{2}\right)-\left(1-\beta \right)}{6000}f,\;0.45<\beta \leq 1 \end{array}$$

We use the expression $\left|1-\beta e^{-2\mathrm{j\pi}f\left(\frac{d}{c}\text{cos}\theta +\tau \right)}\right|$, the dipolar-based parameter τ = 0, and the actual CI parameters d = 0.01 m and c = 340 m/s to calculate the values of H_2_ and H_3_ and to obtain the linear expression of the f-H function, given by Eq. (7).

(7) $$H_{\text{eva}}\left(\beta ,\;f\right)=\{\;\begin{array}{cc} 1-\beta +\left(1.279\times 10^{-4}\times \beta \right)f,\; & \;0\leq \beta \leq 0.45\\ 1-\beta +\left(2.145\times 10^{-4}\times \beta -3.896\times 10^{-5}\right) & \;f,\;0.45<\beta \leq 1 \end{array}$$

**Figure 9:**
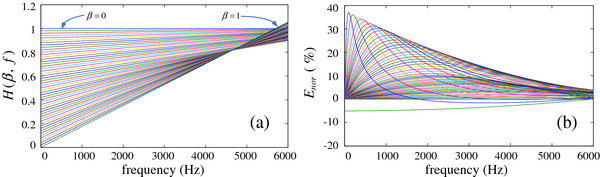
**Linearization curves of the *f*-*H *response function (a) and corresponding errors (b).**

For a fixed beam pattern, we use Eq. (7) to obtain the linear system response. The corresponding response curves are shown in Figure 9 (a).

Panels (a) and (b) present the linear f-H function and the relative errors calculated by Eq. (8), respectively.

(8) $$E=\frac{H_{\text{eva}}\left(\beta ,\;f\right)-H\left(\beta ,\;f\right)}{H\left(\beta ,\;f\right)}\times 100\%$$

As seen in Figure 9(b), the relative error ranges from -10% to 40%. A comparison of Figures 8 and 9(a) indicates that the theoretical f-H function (ideal system response) is primarily a set of concave curves, such that most of the response amplitudes in the linearization curve are greater than the ideal ones. Therefore, the concave feature of the linearization curve indicates that most of the relative errors are positive, but negative relative errors are lacking. Therefore, the whole response amplitudes are larger than the ideal ones. This finding implies that the total energy of the output signal is strengthened.

We only need to analyze the relative amplitude difference for different signal bands because the overall and coordinative enhancement for the compensation will not introduce distortion to the desired signal. To evaluate the compensation error in a detailed and accurate manner, we normalize the relative error to balance the input and output signals. The normalized error is defined by Eq. (9).

(9) $$E_{\text{nor}}=\frac{H_{\text{eva}}\left(\beta ,\;f\right)-G\left(\beta ,\;f\right)\cdot H\left(\beta ,\;f\right)}{H\left(\beta ,\;f\right)}\times 100\%$$

where the normalized coefficient G (β, ƒ) is given in Eq. (10) to maintain the energy equilibrium for a signal between 0 and 6000 Hz. When N is large, the frequency band is fractionized enough, and the calculated normalized coefficient can approximate the ideal value.

(10) $$G\left(\beta ,\;f\right)=\sqrt{\underset{N\to \infty }{\text{lim}}\frac{\sum_{i=0}^{N}{\left|H\left(\beta ,\;\frac{i}{N}\times 6000\right)\right|}^{2}}{\sum_{i=0}^{N}{\left|H\left(\beta ,\;\frac{i}{N}\times 6000\right)|{}_{\text{eva}}\right|}^{2}}}$$

**Figure 10:**
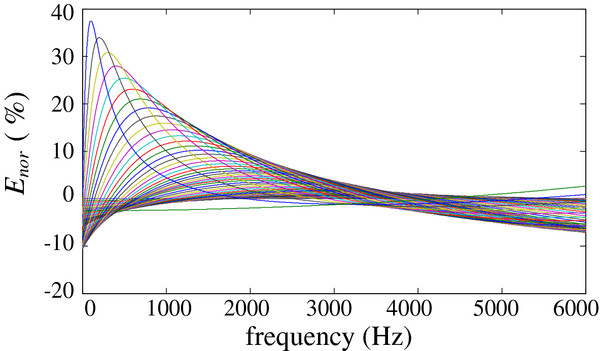
**Normalized errors of the *f*-*H *linearization curves.**

The corresponding normalized error is presented in Figure 10.

According to Figure 10, the normalized error ranges between -10% and 40%. However, most of these errors are concentrated in the ±10% range (corresponding to the error distribution in the high-density dark part of the figure), with only a few large errors. Therefore, most of the errors are very small. Based on a fixed weight parameter, figure 10 also indicates that the error will change for different frequencies.

To evaluate the total error further, we use equation (11) to calculate the average error.

(11) $$E_{\text{ave}}=\lim_{N\to \infty }\frac{1}{N}\sum_{i=1}^{N}\left|E_{\text{nor}|f_{i}|}\right)$$

**Figure 11:**
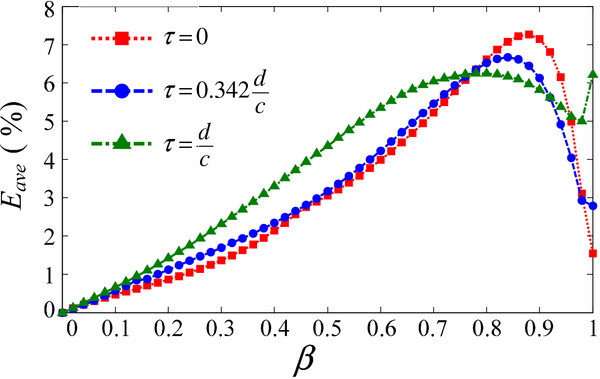
Average errors based on different weights and delay times.

For different weight parameters, the corresponding average error (in red) is shown in Figure 11 (N is 50 in this paper).

For different weight values, the average error (in red) ranges between 0% and 7%, corresponding to the dipolar-based parameter τ = 0. For other delay values, supercardioid- (blue), and cardioid-based (green) parameters, analogous results can be obtained, with average errors ranging from 0% to 7%. These errors are acceptable and sufficiently small for the CI application. For a set of fixed parameters, the system beam pattern is fixed. The proposed linear algorithm for the compensation of low frequency roll-off can approximate the ideal system response with few errors. Additionally, this algorithm is low-calculation for the real-time processing in the CI speech processor.

The broadband signal is divided into several sub-bands during signal processing of the CI speech strategy. Each sub-band includes the signal in its corresponding frequency band, in which the signal envelope information is extracted and transferred to the next process. Because the CI filter bank divides the signal into many bands, the divided signal in each band is approximately narrowband. The CI speech strategy can modulate the information in each band and send it to the electrode array that, with a specific stimulating rate, stimulates the auditory nerve to generate acoustical sensation. The stimulating rates in the electrode array correspond to the center frequencies of the filter bank. The proposed algorithm uses the center frequency f_cen-i_ in each sub-band for the compensation. One of the center frequencies is applied as the reference frequency, f_cen-ref_. Because most speech signals are concentrated in the low-frequency band peaking around 1000 Hz, the reference frequency is chosen in the band near 1000 Hz. For compensation of low frequency roll-off in the i-channel, the corresponding center frequency f_cen-i_ and reference frequency f_cen-ref_ are used in the gain adjustment, as shown in Eq. (12).

(12) $$G_{\text{channel}\;i}=\frac{H_{\text{eva}}\left(\beta ,\;f_{\text{cen}-\text{ref}}\right)}{H_{\text{eva}}\left(\beta ,\;f_{\text{cen}-i}\right)}$$

The reference and i-channel center frequencies in this gain compensation equation are based on the linearization response function; therefore, the gain adjustment needs only a few additional calculations.

### Compensation results in the dual-channel CI platform

**Figure 12:**
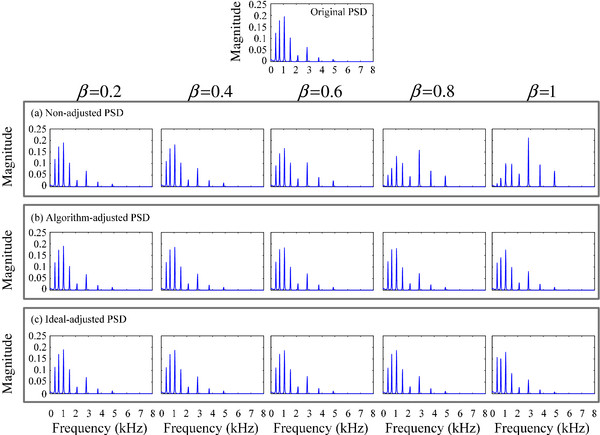
PSD for the CIS signal (a) without adjustment or with adjustment by (b) the proposed algorithm or (c) ideal coefficient for low frequency roll-off.

The low frequency roll-off experiments are conducted on our dual-channel hardware platform. We add compensation gains to the filter bank of the CI speech strategy to adjust the amplitude for each channel. Recorded signals are modulated by the Continuous Interleaved Sampling (CIS) strategy [32]. We use sinusoidal modulation to actualize this speech strategy. A set of sinusoidal signals are modulated by the corresponding envelope of the band-pass signals after frame-division in the filter bank. Then, the original continuous spectrum becomes several discrete frequency components (line spectrum), each of which corresponds to the sub-filter and CI electrode. The electrode array, based on the corresponding stimulating rate, stimulates the nerve to yield auditory perception. In this paper, the frequency components after CIS modulation match the center frequency of the CI filter bank. The Welch method [33] is used to calculate the Power Spectral Density (PSD), to compare results with and without algorithm compensation, as shown in Figure 12.

The experiment is conducted in quiet environment (The SNR is about 15 dB and the performance of the compensated beamformer is evaluated for different noise levels in the discussion section). For different weight parameters, Figure 12 describes the signal PSD (8 line spectrums, corresponding to the 8-channel filter bank) after CIS modulation of the CI device. The top panel presents the original CIS spectrum obtained by an omnidirectional microphone without signal distortion. Figure 12 shows the corresponding CIS spectrums with (b) and without (a) algorithm compensation when the signal is recorded by the dual-channel array. Both CIS spectrums are normalized to balance the overall energy with the original signal. Without compensation of the low frequency roll-off, the signal PSD distribution changes noticeably. The low-frequency amplitude is weakened, whereas the high-frequency amplitude is relatively strengthened for both weight parameters. A greater weight results in more obvious low frequency roll-off. Panel (b) shows that, after algorithm compensation, amplitudes in these 8 channels match the original CIS spectrum well. Therefore, the proposed adjustment algorithm can accurately compensate the signal distortion in array beamforming.

For further comparison, Figure 12(c) shows the compensation results based on the ideal compensation coefficients from the theoretical system response. The ideal compensation uses the i-channel center frequency f_cen-i_ and reference frequency f_cen-ref_ to obtain the gain adjustment, given in Eq. (13).

(13) $$G_{\text{Ideal}-\text{channel}\;i}=\frac{{\left|H\left(e^{j\omega }\right)\right|}_{\beta ,\;f_{\text{cen}-\text{ref}}}}{{\left|H\left(e^{j\omega }\right)\right|}_{\beta ,\;f_{\text{cen}-i\;}}}$$

Equation (2) use the accurate response amplitude, based on a set of fixed frequency and weight parameters, to calculate the ideal adjustment coefficients for the compensation of low frequency roll-off.

**Figure 13:**
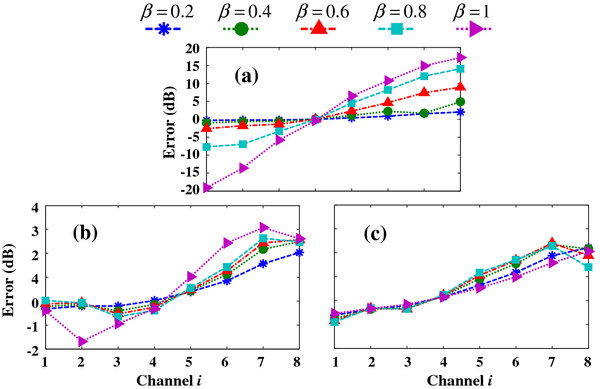
PSD errors for the (a) non-adjusted, (b) algorithm-adjusted, and (c) ideal-adjusted CIS signal, compared to the original CIS signal (in dB).

A comparison of panels (b) and (c) shows that the compensation results by our algorithm are very consistent with the ideal response-based gain adjustment. For detailed comparison, we calculate the corresponding 8-channel spectrum amplitudes for situations (a) without compensation, (b) with compensation by the proposed algorithm, and (c) with ideal compensation. The spectrum amplitudes are compared to the original signal spectrum amplitude (in dB) to obtain the relative enhancement or attenuation results (Figure 13).

Figure 13 shows that low frequency roll-off is obvious when the array output signal is not adjusted (a). The low-frequency signal is weakened, ranging from 0 to -20 dB, and the high-frequency signal is relatively enhanced, ranging from 0 to +20 dB. For the whole frequency band, the signal overall distortion is extremely large, between -20 and +20 dB. Panel (b) presents the compensation results obtained with our proposed algorithm. The signal distortion is very small, with only a few amplitude differences between -2 and 3 dB. When the signal is compensated by the ideal coefficients (c), the error ranges from -1 to 2.5 dB. Therefore, the compensation accuracy by our algorithm can approach the ideal response-based adjustment. The adjusted signal matches the desired CIS spectrum well, with little distortion.

## Discussions

In the previous hardware experiments, the loudspeaker is 1.5 m apart from the hardware. Actually, a common rule of for the range at which the transition from spherical waves to planes waves occurs for a monopole source is at least two times the wavelength, so the corresponding minimal distances for 8 channel of CI filter bank is given in Table 2.

**Table 2:** Minimal distance for 8-channel CI filter bank

	**Center frequency (Hz)**	**Distance between loudspeaker and microphone (m)**
Channel 1	274	2.48
Channel 2	517.5	1.34
Channel 3	761	0.89
Channel 4	1005	0.68
Channel 5	1462	0.47
Channel 6	2376	0.29
Channel 7	3869	0.18
Channel 8	6276	0.11

Seen from Table 2, we find that signal acquisition will be influenced in Channel 1 as the minimal distance Distance between loudspeaker and microphone is 2.48 m. however, the practical usage and daily face-to-face communication for CI users is 1.5 m, so we finally adjusted the loudspeaker 1.5 m apart from the hardware platform. And 1.5 m correspond to 453 Hz, therefore, signal with frequency higher than 453 Hz will not be influenced.

**Figure 14:**
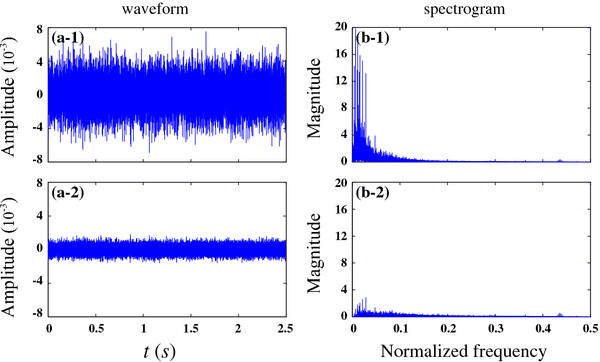
Waveform of (a-1) original background noise, (a-2) first-order differential output signal, and the corresponding spectrum (b-1) and (b-2) respectively.

The environment noise is different from the white noise, with more noise in the low frequency band. In daily usage, the car & fan noise (low-frequency signal) were recorded in microphones. We use the hardware platform to record the environment noise, shown in Figure 14.

Observed from panel (b-1), most environmental noises contain most of the energy concentrated at low frequencies. Panel (b-2) presents the signal spectrum after a first-order differential processing in the dual-microphone array. The corresponding low frequency energy was sharply weakened; however, the amplitude was still very large. Therefore, the low frequency roll-off was not always a bad thing and boosting up low frequency contents might increase internal microphone noise together. A balance between low-frequency distortion and corresponding low-frequency noise need to be considered. This compromise needs an actual test of a set of suitable attenuation coefficient based on omnidirectional microphone, and the coefficients were added in the usage of array beamforming, to obtain low distortion and less low frequency noise.

**Figure 15:**
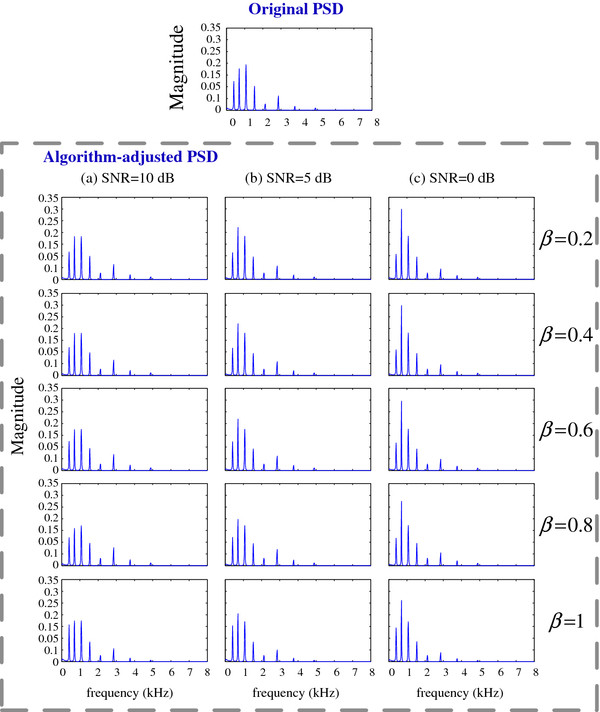
PSD of the CIS signal with the compensated beamformer for different noise levels (a) SNR = 10 dB, (b) SNR = 5 dB and (c) SNR = 0 dB.

For different noise levels (10, 5 and 0 dB respectively), the performance of the compensated beamformer is given in Figure 15.

Figure 15 describes the signal PSD for different noise levels. When noise increase, with smaller SNR, more low-frequency energy was added in. Particularly, the case of SNR = 0 dB presents noticeable results that the signals in low-frequency are enhanced from channel 1 to channel 3. Seen from these panels, lower SNR will introduce more distortion in CI devices.

## Conclusion

The microphone array noise-suppression method can separate the desired speech and ambient noise on the basis of their spatial differences. Use of a dual-channel array with appropriate size constraints is more suitable for CI devices. However, direct application of the narrow-band method in broadband speech will yield low frequency roll-off and noticeable signal distortion. Low-frequency loss from the speech signal can be observed in first- and second-order differential systems [34]. To compensate for low frequency roll-off, conventional methods use only a simple low-pass filter to enhance the low-frequency signal and weaken the high-frequency signal. These methods are not sufficiently accurate to match the original signal. The broadband beamformer was recently introduced as a method to obtain precise compensation. However, these algorithms require extensive calculation, preventing their actual application in CI devices.

In our previous work, we construct a microphone array based platform for signal acquisition. To suppress the environmental noise, we use delay-and-subtract method and proposed the optimal parameter section methods of delay and beamforming for CI speech enhancement [24]. In our later work, we aim to compensate the low frequency roll-off in speech application and proposed the normalized beamforming algorithm using a continuous interleaved sampling strategy [30]. However, this work only contain delay parameter, but without weight parameter. In this paper, we propose a novel CI filter bank-based algorithm for the compensation of low frequency roll-off. This method, with adjustable delay and weight parameters, uses a linear function to approximate the desired system response, with very low computational complexity. Theoretical and experimental results indicate that our algorithm can accurately compensate the signal distortion and is easy to embed in the CI speech strategy, supporting its practical application in the CI device.

## Abbreviations

CI: Cochlear implant; SNR: Signal to noise ratio; CIS: Continuous interleaved sampling strategy.

## Competing interests

The authors declare that they have no competing interests.

## Authors’ contributions

“YC initiated and conceived the algorithm, designed experiments and analyzed the data. QG is the corresponding author. This study was solved under QG’s direction. QG drafted this paper’s manuscript, including content and overall arrangement. QG was also responsible for revising this manuscript. All authors read and approved the final manuscript.”
